# Comparing the Generalizability of Multiregional versus Locally Trained Deep Learning Models for Trachoma Detection

**DOI:** 10.1016/j.xops.2026.101184

**Published:** 2026-04-06

**Authors:** Hady Yazbeck, Jad F. Assaf, Lillian Wheary, Phit Upaphong, Radwa Elsharawi, John Jackson, Dionna M. Wittberg, Scott D. Nash, Fisseha Admassu, Zerihun Tadesse, Solomon Aragie, Ahmed M. Arzika, Andres G. Lescano, Evelyn R. Munayco, John M. Nesemann, Sandra L. Talero, Thomas M. Lietman, Jeremy D. Keenan, Travis K. Redd

**Affiliations:** 1 Casey Eye Institute, Oregon Health & Science University, Portland, Oregon; 2 College of Osteopathic Medicine of the Pacific-Northwest, Western University of Health Sciences, Lebanon, Oregon; 3 Francis I Proctor Foundation and Department of Ophthalmology, University of California, San Francisco, California; 4 The Carter Center, Atlanta, California; 5 Department of Ophthalmology, University of Gondar, Gondar, Ethiopia; 6 The Carter Center, Addis Ababa, Ethiopia; 7 The Carter Center, Niamey, Niger; 8 Centre de Recherche et Interventions en Santé Publique (CRISP), Birni N'Gaoure, Niger; 9 Emerging Diseases and Climate Change Research Unit (Emerge), School of Public Health and Administration, Universidad Peruana Cayetano Heredia, Lima, Peru; 10 Pan American Health Organization (PAHO), Washington, DC

**Keywords:** Trachoma, Artificial intelligence, Generalizability, Deep learning, Geographic diversity

## Abstract

**Objective:**

To test the advantage of geographically diverse, multiregional training of artificial intelligence models over single-region training for detection of trachomatous inflammation-follicular (TF) across test sets from different regions.

**Design:**

A comparative study evaluating the performance of trachoma detection models trained on single-region image datasets versus multiregional datasets.

**Subjects:**

A total of 71 206 everted eyelid photographs from 15 605 subjects aged from 0 to 9 years.

**Methods:**

Everted eyelid photographs from Ethiopia, Niger, and Peru were collected between 2014 and 2020. They were graded for the presence or absence of TF by certified experts. The resulting labels were used to train, validate, and test 3 single-region models and multiregional models for trachoma detection.

**Main Outcome Measures:**

The F1-score, area under the receiver operating characteristic curve (AUROC), and predicted TF prevalence were used to measure performance of all models on each of the test sets from Ethiopia, Niger, and Peru. Heatmaps were generated to visualize the highest areas of activation.

**Results:**

Results are reported for the Ethiopia, Niger, and Peru test sets in that order. Except for Niger, single-region models performed best on their respective local test sets with F1 = 0.78; 0.24; 0.89 and AUROC = 0.94; 0.97; 0.98, and accurate prevalence predictions. However, they performed significantly worse on data from other regions. In contrast, the multiregional model accurately estimated trachoma prevalence in all test sets across all regions and matched the performance of the single-region models on their respective test sets (F1 = 0.85; 0.25; 0.89 and AUROC = 0.96; 0.79; 0.99). Heatmaps showed agreement with diagnostically relevant trachoma features.

**Conclusions:**

Geographically diverse training data are essential to developing broadly generalizable deep learning models for trachoma detection. Using such models to guide mass distribution of macrolide antibiotics may improve the scalability and cost effectiveness of trachoma control campaigns.

**Financial Disclosure(s):**

Proprietary or commercial disclosure may be found in the Footnotes and Disclosures at the end of this article.

Trachoma, still the most common infectious cause of blindness worldwide, is caused by the bacterium *Chlamydia trachomatis*.^1^ Infected ocular and nasal secretions are spread through fingers, flies, and fomites.^2^ Clinically, active infection presents as conjunctivitis characterized by follicular and papillary inflammation. The World Health Organization (WHO) created a simplified grading system for trachoma, with the key inflammatory indicators being trachomatous inflammation-follicular (TF) and trachomatous inflammation-intense.^3^

The WHO recommends population-based prevalence surveys to guide public health decision-making for trachoma. A key indicator is the prevalence of TF among children aged 1 to 9 years as assessed by field graders.^4^ Districts with a TF prevalence of ≥5% are recommended to receive mass distribution of macrolide antibiotics, and elimination is not declared until all districts in a country have a TF prevalence <5%.^5^

The current paradigm of field examination for trachoma faces several challenges. In-person examinations are inherently subjective and subject to considerable intragrader and intergrader variabilities, compromising the accuracy of prevalence estimates.^6^ Maintaining a well-trained field grading staff throughout an entire trachoma-endemic country is logistically challenging and expensive. Moreover, experienced field graders are not available in some places, especially in settings with low-trachoma prevalence.^7^ Therefore, a more scalable, sustainable, and cost-effective trachoma surveillance method would be useful.

Over the past decade, the rise of deep learning and convolutional neural networks (CNNs) have revolutionized image-based diagnostics, demonstrating human-level image-interpretation capabilities across a broad range of tasks.^8^ In trachoma detection specifically, our prior work has demonstrated that deep CNNs are capable of image-based detection of TF with high accuracy.^9^ However, medical artificial intelligence (AI) models often fail to generalize to populations that are not well represented in their training data. Examples of this phenomenon have been reported across different fields, including ophthalmology, cardiovascular disease, radiology, and dermatology.^10^, ^11^, ^12^, ^13^, ^14^, ^15^, ^16^ Moreover, a recent report in 2021 noted that 172 countries (totaling 3.5 billion people) have no publicly available ophthalmic imaging datasets, further highlighting the risk of sampling bias and subsequent poor generalizability to the populations of those countries.^17^ There is evidence in ophthalmology showing better generalizability of AI models in detecting retinopathy of prematurity when trained on more diverse datasets,^18^ but to our knowledge, this approach has not yet been investigated in trachoma. Additionally, because glaucoma, diabetic retinopathy, and age-related macular degeneration represent the majority of publicly available datasets,^17^ there is a need to advance research in other less represented eye diseases, especially when they carry as high a public health burden as trachoma. In this study, we assess the generalizability of deep learning models trained to detect trachoma by comparing the performance of models trained on photographs from a single country (single-region models) versus models trained on photographs from 3 different countries (multiregional models). In the process, we trained and validated a model for automated TF detection on the largest and most diverse set of conjunctival images yet reported in the literature.

## Methods

This retrospective study was conducted at the Casey Eye Institute, Oregon Health & Science University and was approved by the Oregon Health & Science University Institutional Review Board. All study procedures complied with the Health Insurance Portability and Accountability Act of 1996 and adhered to the principles of the Declaration of Helsinki. Informed consent was waived by the institutional review board because the analysis used data obtained from prior studies for which informed consent had already been obtained, and no additional participant contact or intervention occurred^19^^,^^20^ (Keenan J, unpublished).

### Literature Search

To examine the current evidence for AI-based detection of TF, we conducted a systematic search of PubMed from inception to October 2, 2025, using the query: ("Trachoma"[MeSH] OR trachoma[Title/Abstract]) AND ("artificial intelligence"[All Fields] OR "machine learning"[All Fields] OR "deep learning"[All Fields] OR "neural network"[All Fields] OR "CNN"[All Fields] OR "computer vision"[All Fields] OR "AutoML"[All Fields]).

### Data Acquisition

We used conjunctival photographs from 3 prior studies conducted in Ethiopia, Niger, and Peru. Images from Ethiopia were obtained as part of the Water, Sanitation, and Hygiene Upgrades for Health in Amhara study, a cluster-randomized trial conducted from 2016 to 2019 in the WagHemra zone of Ethiopia.^19^ The age range in this dataset was 0 to 9 years, with more detailed demographic information outlined in the study.^19^ Images were collected using a Samsung Galaxy NX camera with a macro lens. Images from Niger came from a trial examining biannual mass azithromycin distributions for trachoma in the Boboye and Loga departments of Niger, conducted from 2014 to 2017 as part of the Macrolides Oraux pour Réduire les Décés Avec un Oeil sur la Resistance (MORDOR) trial.^20^ The population in this dataset was 47.3% female, and the age range was 1 to 59 months with mean ± standard deviation of 34.64 ± 17.81 months. These images were captured using a Google Nexus 5 with a CellScope attachment.^21^ The Peru image dataset came from a 2020 study assessing clinical trachoma and chlamydial infection in the Alto Amazonas province of Peru (Keenan J, unpublished). The population in this dataset was 49.6% female, and the age range was 1 to 9 years with mean ± standard deviation of 5.05 ± 2.54 years. Data were collected using a Xiaomi A2 smartphone equipped with CellScope. All images were evaluated for the presence of TF using the WHO simplified grading system^3^ by ≥2 trained and certified graders. Discrepancies were resolved by a third independent grader. The majority consensus TF grade was used as the ground truth label for model training and evaluation. Ethical approval was obtained from institutional review boards at the University of California, San Francisco, the Ethiopian Ministry of Science and Technology, the Food, Medicine, and Healthcare Administration and Control Authority of Ethiopia, the Nigerien Ministry of Health, the Peruvian Ministry of Health, and Universidad Peruana Cayetano Heredia.

### Single-Region Datasets

To enable equitable performance comparisons among the different single-region models, the number of subjects in each dataset was restricted via random sampling to that of the smallest dataset, which consisted of 767 participants from the Peru study. The total number of images was also restricted to comparable numbers across the 3 datasets. As a result, the Ethiopia dataset consisted of 2314 images from 767 participants, with about 29% TF prevalence according to the expert graders. The Niger dataset included 2278 images from 767 children with approximately 3% prevalence of TF. The Peru dataset contained 2475 images from 767 children with a TF prevalence of about 26%.

### Multiregional Datasets

To compare the impact of multiregional versus single-region training data, we developed a multiregional dataset of the same size as each of the single-region datasets by randomly sampling one-third of the cases from each regional dataset. This resulted in around 2300 images from 767 patients. We termed this the “limited multiregional dataset.” The purpose of this dataset was to match the training and validation sizes of the single-region datasets, allowing analysis of the potential benefits of multiregional training without having dataset size confounding the comparison.

To assess the added value of increasing the training and validation set sizes, we established a second multiregional dataset (the “complete multiregional dataset”) consisting of all the available cases from each region, totaling 71 206 images from 15 605 participants (56 483 photographs from 11 358 children in Ethiopia; 10 305 photographs from 3480 children in Niger; 4418 photographs from 767 children in Peru). This constitutes the largest and most diverse set of conjunctival images used to train a deep learning model yet reported in the literature.

### Model Architecture and Image Processing

We used a MobileNetV3Large deep CNN architecture with pretrained weights from ImageNet.^22^^,^^23^ The reason for our choice is the relatively small size (4.2 million parameters) of the model, which facilitates implementation on portable devices such as smartphones. This potentially enables a more straightforward integration into existing trachoma screening programs for maximal impact. Images were resized to 224 × 224 pixels using bilinear interpolation and normalized for input into the CNN. Data augmentation during training included random flips, rotations, hue/brightness/contrast changes, and random location patch noise induction. Training was performed with 2 NVIDIA GeForce RTX 4090 GPUs on a Lambda Vector workstation (Lambda Labs) using Python (version 3.10.12, Python Software Foundation) and PyTorch (version 2.5.1).

### Training Strategy

For all datasets, data from 20% of the participants was allocated for testing, and the remaining 80% was used for training and validation via fivefold cross-validation. All splits were performed at the participant level to avoid label leakage. Detailed information on the training, validation, and test dataset composition for all single-region and multiregional models can be found in the Supplementary Material (Tables 2–4, available at www.ophthalmologyscience.org). We then trained 3 single-region models; one for each regional dataset, and 2 multiregional models; one for the limited multiregional dataset, and one for the complete multiregional dataset. We used a batch size of 128, a discriminative learning rate following the OneCycle policy, and the Adam optimizer.^24^^,^^25^ Dropout and weight decay were optimized as needed. Binary cross entropy with logits loss was used as the criterion. All information on model hyperparameters can be found in the Supplementary Material (available at www.ophthalmologyscience.org).

The source code for model training, loading, and evaluation is freely available on GitHub at https://github.com/Redd-Cornea-AI/Trachoma-detection-from-smartphone-images-using-multi-regional-centralized-training/tree/main/Scripts.

### Evaluation Metrics and Statistical Analysis

**Figure 1:**
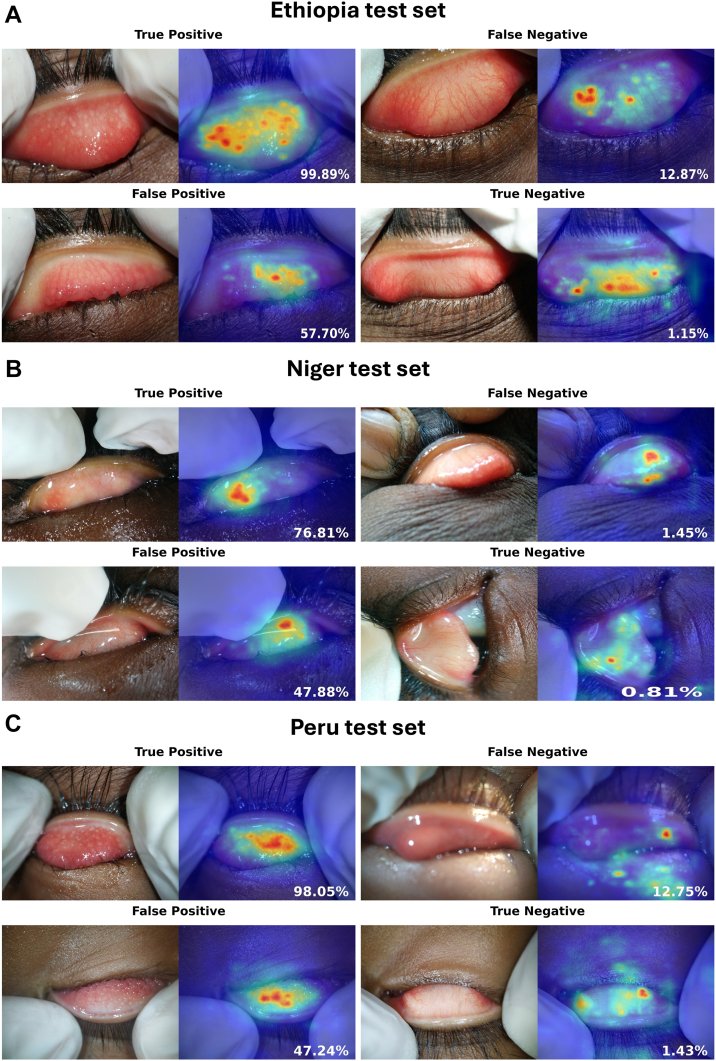
LayerCAM heatmaps with model's predictions. The trachoma detection threshold is 27%. Examples are shown for TP, FN, TN, and FP predictions across **(A)** the Ethiopia test set, **(B)** the Niger test set, and **(C)** the Peru test set. Most TP cases show focus in areas of trachomatous follicles, while FNs were some borderline cases with subtle follicles and prominent vascular pattern (Ethiopia) or instances of out-of-focus images (Peru, Niger). True negative cases seemed to delineate the model's focus on the vascular pattern of the conjunctiva. False positive cases included vernal kerato-conjunctivitis (Ethiopia, Peru) and flashlight reflection (Niger). FN = false negative; FP = false positive; TN = true negative; TP = true positive.

In each case, the best-performing (lowest validation loss) fold during cross-validation was selected as the optimal model, which was then evaluated on the corresponding holdout test set. Because subjects had multiple images per eye, the predictions for images on the test set were averaged to obtain a single subject-level prediction during inference. Model predictions were then binarized using the optimal threshold obtained by calculating the Youden index on the validation set (i.e., the threshold maximizing the sum of sensitivity and specificity on the validation set).^26^ Three metrics were used to measure model performance on each test set: F1-score (Table 1), area under the receiver operating characteristic curve (AUROC) (Table 2), and prevalence estimation (Table 3). For the prevalence estimation metric, all models were compared to the human-derived estimate. To assess the statistical significance of performance differences, we performed a nonparametric bootstrap test with 10 000 resampling iterations to compute 95% confidence intervals and 2-sided *P* values. A significance threshold of 0.05 was used and adjusted for multiple comparisons using the Bonferroni correction. Finally, we employed LayerCAM^27^ heatmaps to visualize regions of highest activation in the best-performing models (Fig 1). Detailed metrics reporting, including area under the precision and recall curve, sensitivity, specificity, precision and accuracy, can be found in the Supplementary Material (available at www.ophthalmologyscience.org).

**Table 1:** Best-Fold Model Performance (F1 Score [95% Bootstrapped CI] Using 10 000 Iterations) across Different Test Sets

	Ethiopia Model	Niger Model	Peru Model	Limited Multiregional Model	Complete Multiregional Model
Ethiopia test set (N = 154)	0.78 [0.68, 0.87]	0.48 [0.39, 0.58] (*P* < 0.001∗)	0.55 [0.38, 0.68] (*P* = 0.002∗)	0.75 [0.64, 0.84] (*P* = 0.44)	0.85 [0.76, 0.92] (*P* = 0.06)
Niger test set (N = 154)	0.11 [0.03, 0.21] (*P* = 0.007∗)	0.24 [0.06, 0.41]	0.30 [0.00, 0.55] (*P* = 0.57)	0.29 [0.00, 0.73] (*P* = 0.88)	0.25 [0.00, 0.67] (*P* = 0.97)
Peru test set (N = 154)	0.43 [0.33, 0.51] (*P* < 0.001∗)	0.66 [0.53, 0.77] (*P* = 0.001∗)	0.89 [0.81, 0.95]	0.84 [0.75, 0.92] (*P* = 0.18)	0.89 [0.81, 0.95] (*P* = 0.87)

CI = confidence interval.

∗ Statistically significant (*P* < significance threshold) difference in performance compared to the single-region model trained and evaluated on the same-region test set. For example, the Niger and Peru models have a statistically significantly lower F1-score on the Ethiopia test set, when compared to the Ethiopia model. The multiregional models do not. Significance threshold is set to 0.05/4 = 0.0125 to correct for multiple hypothesis testing.

**Table 2:** Best-Fold Model Performance (AUROC ± 95% Bootstrapped CI Using 10 000 Iterations) across Different Test Sets

	Ethiopia Model	Niger Model	Peru Model	Limited Multiregional Model	Complete Multiregional Model
Ethiopia test set (N = 154)	0.94 [0.88, 0.98]	0.73 [0.65, 0.82] (*P* < 0.001∗)	0.82 [0.74, 0.90] (*P* = 0.006∗)	0.90 [0.84, 0.95] (*P* = 0.06)	0.96 [0.92, 0.99] (*P* = 0.09)
Niger test set (N = 154)	0.80 [0.61, 0.93] (*P* = 0.04)	0.97 [0.93, 1.00]	0.82 [0.56, 0.98] (*P* = 0.14)	0.81 [0.57, 0.98] (*P* = 0.09)	0.79 [0.48, 0.99] (*P* = 0.10)
Peru test set (N = 154)	0.76 [0.67, 0.85] (*P* < 0.001∗)	0.84 [0.77, 0.91] (*P* < 0.001∗)	0.98 [0.96, 0.99]	0.97 [0.94, 0.99] (*P*= 0.29)	0.99 [0.97, 1.00] (*P* = 0.11)

AUROC = area under the receiver operating characteristic curve; CI = confidence interval.

N represents the number of subjects in each test set.

∗ Statistically significant (*P* < significance threshold) difference in performance compared to the single-region model trained and evaluated on the same-region test set. For example, the Niger and Peru models have a statistically significantly lower AUROC on the Ethiopia test set when compared to the Ethiopia model. The multiregional models do not. Significance threshold is set to 0.05/4 = 0.0125 to correct for multiple hypothesis testing.

**Table 3:** Prevalence Estimates (in %) of Best-Fold Model (95% Bootstrapped CI Using 10 000 Iterations) versus Human Estimate ([Positive Cases/Total Cases] × 100) across Different Test Sets

	Human Estimate	Ethiopia Model	Niger Model	Peru Model	Limited Multiregional Model	Complete Multiregional Model
Ethiopia test set (N = 154)	28.57 (44/154)	34.42 [26.62, 42.21] (*P* = 0.06)	75.97 [68.83, 82.47] (*P* < 0.001∗)	14.29 [9.09, 20.13] (*P* < 0.001∗)	31.82 [24.68, 39.61] (*P* = 0.32)	32.47 [25.32, 40.26] (*P* = 0.13)
Niger test set (N = 154)	3.25 (5/154)	54.55 [46.75, 62.34] (*P* < 0.001∗)	24.03 [17.53, 31.17] (*P* < 0.001∗)	9.74 [5.19, 14.29] (*P* = 0.007∗)	1.30 [0.00, 3.25] (*P* = 0.25)	1.95 [0.00, 4.55] (*P* = 0.53)
Peru test set (N = 154)	25.97 (40/154)	96.10 [92.86, 98.70] (*P* < 0.001∗)	29.22 [22.08, 36.36] (*P* = 0.37)	27.92 [20.78, 35.06] (*P* = 0.36)	31.17 [24.03, 38.31] (*P* = 0.03)	31.17 [24.03, 38.31] (*P* = 0.014)

CI = confidence interval.

N represents the number of subjects in each test set.

∗ Statistically significant (*P* < significance threshold) difference compared to the human estimate on a given test dataset. For example, in the Ethiopia test set, the Peru and Niger models' prevalence estimation significantly differed from the human estimate, while that of the Ethiopia and multiregional models did not. Significance threshold is set to 0.05/5 = 0.01 to correct for multiple hypothesis testing.

## Results

Single-region models achieved the highest F1 scores when evaluated on the test set from the same region they were exclusively trained on (F1 = 0.78, 0.24, and 0.89 for Ethiopia, Niger, and Peru, respectively), but performed statistically significantly worse on the test sets from the other regions (Table 1). When tested on the Niger dataset, only the Ethiopia-trained model performed significantly worse than the Niger-trained model (F1 = 0.11 vs. 0.24). Both the limited and complete multiregional models exhibited similar performance compared to that of the single-region models on their respective local test sets (limited model F1 = 0.75, 0.29, and 0.84 and complete model F1 = 0.85, 0.25, and 0.89 on Ethiopia, Niger, and Peru, respectively). A similar pattern was observed for AUROC (Table 2), with single-region models showing the highest performance on their respective regional test sets (AUROC = 0.94, 0.97, and 0.98 for Ethiopia, Niger, and Peru, respectively), and multiregional models matching that performance across all datasets (limited model AUROC = 0.90, 0.81, and 0.97 and complete model AUROC = 0.96, 0.79, and 0.99 on Ethiopia, Niger, and Peru, respectively).

With respect to prevalence estimation results, shown in Table 3, a model's prediction on a specific regional test set was compared to the human prevalence estimate on conjunctival photographs from that same region. The human-derived prevalence estimates were 28.57%, 3.25%, and 25.97% on the Ethiopia, Niger, and Peru test sets, respectively. The prevalence prediction from the Ethiopia and Peru-trained models on their respective test sets (34.42% and 27.92%, respectively) agreed with the human estimate, but disagreed with that of test sets from different regions. The prevalence prediction from the Niger-trained model on its respective test set (24.03%) disagreed with the human estimate. It also disagreed with the human prevalence estimate on Ethiopia, but agreed with that of Peru. The prevalence estimates of the limited multiregional model (31.82%, 1.30%, and 31.17%) and complete multiregional model (32.47%, 1.95%, and 31.17%) on the Ethiopia, Niger, and Peru test sets agreed with the human estimates with no statistically significant differences.

Interpretability analysis was performed for the complete multiregional model using LayerCAM heatmaps, which appeared consistent with diagnostically relevant features of TF. In true positive cases, the model seemed to focus on regions in the everted tarsal conjunctiva containing trachomatous follicles. It appeared to us that true negative cases seemed to delineate activation areas around the vascular pattern of the conjunctiva. Among false negatives, several were borderline cases with subtle follicles with a prominent vascular pattern, or instances of improperly exposed or out-of-focus conjunctiva. In false positive cases, the most notable findings were borderline cases, flashlight reflections, and cases of vernal keratoconjunctivitis. Representative examples are shown in Figure 1A–C.

## Discussion

The aim of this study was to compare multiregional training of AI models to single-region training for automated detection of TF, a preventable, leading infectious cause of blindness. We also proceed to train and validate a model on the largest and most diverse set of conjunctival images yet reported in the literature. This is important because models trained on large and geographically diverse datasets may have the potential to improve cross-regional generalizability in trachoma prevalence estimation, which can better guide mass antibiotic distribution in endemic areas and control the disease.

Overall, our findings reveal that single-region models, while highly performant on their respective local data, often show diminished performance on external datasets from other regions. These results suggest the presence of local features that may bias single-region models to rely on patterns that are poorly generalizable across different populations. Our findings are consistent with the broader machine learning literature. It has long been a concern that medical AI models may underperform when applied to external datasets of different patient populations, races, or ethnicities.^10^, ^11^, ^12^, ^13^ For example, in diabetic retinopathy detection, a deep learning model trained on color fundus photographs exhibited wide variation in diagnostic accuracy for light-skinned versus dark-skinned individuals (73% vs. 60.5%, respectively).^14^ This was attributed to phenotypic factors such as increased retinal pigmentation, darker skin tone, optic disc size, and retinal vessel caliber.^14^^,^^28^ By extension, poor generalizability of trachoma detection across geographically different populations may stem from differences in ocular pigmentation, eyelid shape, conjunctival vascular pattern, or even features that are not recognizable to the human eye.^29^^,^^30^ Socia et al’s^11^ study results showed that a trachoma detection model trained on a dataset from Tanzania poorly generalized to a test set from Niger and Ethiopia, and vice versa. However, they did not test the multiregional training approach to mitigate this problem, as is done in this study.

Beyond cross-regional generalizability issues, different imaging conditions or environmental cues may also affect model predictions. For example, in a 2018 study examining CNNs for pneumonia detection on chest X-rays from 3 hospitals in the United States, Zech et al^31^ found that the CNNs were able to recognize, with extremely high accuracy, the hospital in which a radiograph was acquired. Attention heatmaps revealed that the models had learned to detect a metal token placed by radiology technicians on the patient at the time of the scan. When these features are correlated with disease prevalence, models can leverage them to indirectly predict disease. Similarly, in trachoma detection, it is possible that certain environmental cues in different imaging centers, or subtle factors like image acquisition differences between smartphones, may bias the model's performance when only trained on that data. Notably, our multiregional models were trained on images acquired across imaging centers in 3 different countries, and using 2 different imaging modalities: one camera and 2 different smartphone models. This diversity may potentially broaden their generalizability across a wider range of imaging devices and conditions.

We also show in our study how combining training and validation data from multiple regions can considerably improve a model's generalizability without sacrificing local performance. This is reflected by the multiregional models' ability to consistently detect trachoma with comparable performance to the single-region model trained on the same-region dataset. Importantly, by restricting training and validation dataset size in the limited multiregional model, we show that this advantage purely stems from geographic diversity in training. It is possible that, in addition to being exposed to data from different regions, the model is forced to learn region-agnostic, generalizable trachoma features that it can use when predicting cases from different regions. On the other hand, the data volume advantage was observed with the performance of the complete multiregional model, which outperformed the limited multiregional model. Such a model for TF detection may have the potential to be deployed in trachoma-endemic regions with limited access to expert graders. By enabling efficient and objective estimation of community-level TF prevalence, it could potentially support the WHO surgery, antibiotics, facial cleanliness, and environmental improvements (SAFE) strategy by identifying areas exceeding the 5% treatment threshold and guiding targeted mass antibiotic administration using highly scalable, inexpensive technology.

A notable finding in our results is that all models performed poorly on the Niger dataset according to the F1-score, with values ranging from 0.11 to 0.30. This can be explained by the extreme data imbalance with negative-class dominance in the Niger dataset, which affects both the training and validation processes. From the training aspect, the overwhelming exposure of the model to negative cases may sway its outputs to the lower, near 0 predictions (i.e., the model does not see enough positive cases and learns to predict almost all cases as negative). In addition, the low prevalence of the positive class reduces the model's robustness to common image quality issues present within that class, such as inadequate conjunctival exposure or blur. Upsampling of the positive class and weighted loss implementation did not significantly improve our results. From the validation and testing aspects, low F1-scores can be explained by looking at the precision component. Namely, precision is especially sensitive to false positive predictions in cases of data imbalance with negative class dominance. The number of positive cases is so low that even a few false positive predictions considerably impact precision. Furthermore, resampling false positives in the bootstrapping process enhances this effect and considerably widens the confidence interval of the F1-score. These combined factors blur statistical significance and make it hard to draw conclusions from the Niger test set, which constitutes a limitation of our study. In contrast to the poor F1-scores, the AUROCs of all models on the Niger dataset were relatively better (≥0.70). This discrepancy is due to the specificity component of AUROC being much less susceptible to false positives compared to precision in cases of negative data imbalance. This is because a disproportionately high number of true negatives can dilute the impact of false positives in the specificity formula. This example shows how different performance metrics can be affected by data imbalance, highlighting the importance of reporting all evaluation metrics of an AI model, especially in cases when predictions impact the health care of patients.

Importantly, while the poor F1-scores of our models on a dataset with extremely low prevalence may raise validity concerns in a near or postelimination setting, the prevalence estimates of the multiregional models (0.01 for the limited model and 0.02 for the complete model) were significantly improved compared with the single-region models and did not significantly differ from the human estimate of 0.03. This finding indicates that, compared with the single-region models, the overall number of misclassified photographs in the multiregional models was in fact considerably reduced and did not materially bias the prevalence estimate.

This study is not without limitations. First, datasets from only 3 regions were included in our study. It would be interesting to further explore the generalizability of our findings to other trachoma-endemic areas. Second, our work focused on TF detection; models detecting other manifestations of trachoma, such as trachomatous inflammation-intense or trichiasis, were beyond our scope and remain to be studied. Third, the applicability of our model on conjunctival images acquired in actual clinical settings, as opposed to a controlled research environment, remains to be assessed. This will be a focus of our future research. Finally, multiregional training was performed via the centralized learning approach, which involves combining multicenter data into a single repository to train a unified model. This approach presents significant challenges related to data privacy in data sharing, which can impede multicenter and international collaborations.^10^ Therefore, it becomes relevant to consider more privacy-preserving technologies, such as federated learning, which transmits only model parameters or gradients instead of raw imaging data, enabling diversity of training while maintaining patient privacy. In our future work, we plan to compare the performance of federated versus centralized learning and validate our complete multiregional model on additional external test sets from different geographic areas.

## References

[1] Knirsch C. (2007). Trachoma: ancient scourge, disease elimination, and future research. Curr Infect Dis Rep.

[2] Solomon A.W., Burton M.J., Gower E.W. (2022). Trachoma Nat Rev Dis Primers.

[3] (2004). WHO simplified trachoma grading system. Commun Eye Health.

[4] Lansingh V.C., Carter M.J. (2007). Trachoma surveys 2000-2005: results, recent advances in methodology, and factors affecting the determination of prevalence. Surv Ophthalmol.

[5] Trachoma. https://www.who.int/news-room/fact-sheets/detail/trachoma.

[6] Gebresillasie S., Tadesse Z., Shiferaw A. (2015). Inter-rater agreement between trachoma graders: Comparison of grades given in field conditions versus grades from photographic review. Ophthalmic Epidemiol.

[7] Habtamu E., Wondie T., Aweke S. (2015). Trachoma and relative poverty: a case-control study. PLoS Negl Trop Dis.

[8] LeCun Y., Bengio Y., Hinton G. (2015). Deep learning. Nature.

[9] Joye A.S., Firlie M.G., Wittberg D.M. (2024). Computer vision identification of trachomatous inflammation-follicular using deep learning. Cornea.

[10] Fiske A., Blacker S., Geneviève L.D. (2025). Weighing the benefits and risks of collecting race and ethnicity data in clinical settings for medical artificial intelligence. Lancet Digital Health.

[11] Socia D., Brady C.J., West S.K., Cockrell R.C. (2022). Detection of trachoma using machine learning approaches. PLoS Negl Trop Dis.

[12] Cau R., Pisu F., Suri J.S., Saba L. (2025). Addressing hidden risks: systematic review of artificial intelligence biases across racial and ethnic groups in cardiovascular diseases. Eur J Radiol.

[13] Nakayama L.F., Kras A., Ribeiro L.Z. (2022). Global disparity bias in ophthalmology artificial intelligence applications. BMJ Health Care Inform.

[14] Burlina P., Joshi N., Paul W. (2021). Addressing artificial intelligence bias in retinal diagnostics. Transl Vis Sci Technol.

[15] Pyrros A., Rodríguez-Fernández J.M., Borstelmann S.M. (2022). Detecting racial/ethnic health disparities using deep learning from frontal chest radiography. J Am Coll Radiol.

[16] Towards Fairness in AI for Melanoma Detection: Systemic review and recommendations. https://arxiv.org/html/2411.12846v1.

[17] Khan S.M., Liu X., Nath S. (2021). A global review of publicly available datasets for ophthalmological imaging: barriers to access, usability, and generalisability. Lancet Digital Health.

[18] Chen J.S., Coyner A.S., Ostmo S. (2021). Deep learning for the diagnosis of stage in retinopathy of prematurity. Ophthalmol Retina.

[19] Aragie S., Wittberg D.M., Tadesse W. (2022). Water, sanitation, and hygiene for control of trachoma in Ethiopia (WUHA): a two-arm, parallel-group, cluster-randomised trial. Lancet Glob Health.

[20] Arzika A.M., Mindo-Panusis D., Abdou A. (2022). Effect of biannual mass azithromycin distributions to preschool-aged children on trachoma prevalence in niger: a cluster randomized clinical trial. JAMA Netw Open.

[21] Nesemann J.M., Seider M.I., Snyder B.M. (2020). Comparison of smartphone photography, single-lens reflex photography, and field-grading for trachoma. Am J Trop Med Hyg.

[22] Howard A, Sandler M, Chu G (2019). Searching for MobileNetV3.

[23] Russakovsky O., Deng J., Su H. (2015). ImageNet large scale visual recognition challenge. arXiv.

[24] Smith L.N., Topin N. (2017). Super-convergence: very fast training of neural networks using large learning rates. arXiv.

[25] Kingma D.P., Ba J. (2017). Adam: a method for stochastic optimization. arXiv.

[26] Youden W.J. (1950). Index for rating diagnostic tests. Cancer.

[27] LayerCAM Exploring hierarchical class activation maps for localization | IEEE Journals & Magazine | IEEE Xplore. https://ieeexplore.ieee.org/document/9462463.

[28] Tan T.F., Teo Z.L., Ting D.S.W. (2023). Artificial intelligence bias and ethics in retinal imaging. JAMA Ophthalmol.

[29] Coyner A.S., Singh P., Brown J.M. (2023). Association of biomarker-based artificial intelligence with risk of racial bias in retinal images. JAMA Ophthalmol.

[30] Gichoya J.W., Banerjee I., Bhimireddy A.R. (2022). AI recognition of patient race in medical imaging: a modelling study. Lancet Digit Health.

[31] Zech J.R., Badgeley M.A., Liu M. (2018). Variable generalization performance of a deep learning model to detect pneumonia in chest radiographs: a cross-sectional study. PLoS Med.

