# Supplementary material for: Comparing the Generalizability of Multiregional versus Locally Trained Deep Learning Models for Trachoma Detection
Source: Ophthalmol Sci. 2026 Apr 6;6(6):101184. doi: 10.1016/j.xops.2026.101184 (PMC13156724; doi:10.1016/j.xops.2026.101184)
Supplement: Supplementary material [file mmc1.docx]

**Supplementary material**

**Table of contents**

1. Model hyperparameters … page 2, 1 table.
   1. **Table 1:** Hyperparameters used for model training. LR = Learning Rate, BCE = Binary Cross Entropy. All models used a MobileNetV3Large deep CNN architecture ^1^ (4.2 million parameters) with pre-trained weights from ImageNet ^2^.
2. Supplemental results … pages 2–4, 6 tables.
   1. **Table 2:** Training and validation dataset composition of the Ethiopia, Niger and Peru single-region models.
   2. **Table 3:** Training and validation dataset composition of the Limited and Complete Multi-regional models. Data are shown as numbers (proportion in %).
   3. **Table 4:** Ethiopia, Niger, and Peru Test set composition. Note that all single-region and multi-regional models were tested on these test sets for fair performance comparison.
   4. **Table 5:** Best-fold Ethiopia-trained model performance (Metric ± 95% bootstrapped CI using 10,000 iterations) across different test sets. Binarization threshold = 0.20.
   5. **Table 6:** Best-fold Niger-trained model performance (Metric ± 95% bootstrapped CI using 10,000 iterations) across different test sets. Binarization threshold = 0.0002.
   6. **Table 7:** Best-fold Peru-trained model performance (Metric ± 95% bootstrapped CI using 10,000 iterations) across different test sets. Binarization threshold = 0.20.
   7. **Table 8:** Best-fold Limited multi-regional model performance (Metric ± 95% bootstrapped CI using 10,000 iterations) across different test sets. Binarization threshold = 0.25.
   8. **Table 9:** Best-fold Complete multi-regional model performance (Metric ± 95% bootstrapped CI using 10,000 iterations) across different test sets. Binarization threshold = 0.27.
3. References … page 4

**Model Hyperparameters**

**Table 1:** Hyperparameters used for model training. All models used a MobileNetV3Large deep CNN architecture ^1^ (4.2 million parameters) with pre-trained weights from ImageNet ^2^. LR: Learning Rate, BCE: Binary Cross Entropy.

|  | **Batch size** | **Epochs** | **LR decay factor** | **LR scheduler** | **Dropout rate** | **Weight**  **decay** | **Criterion** | **Positive class weight** | **Optimizer** |
| --- | --- | --- | --- | --- | --- | --- | --- | --- | --- |
| Ethiopia model | 128 | 50 | 0.1 | OneCycleLR ^3^ | 0.2 | 0.05 | BCE loss | 1.0 | Adam ^4^ |
| Niger model | 128 | 50 | 0.01 | OneCycleLR | 0.3 | 0.05 | BCE loss | 1.5 | Adam |
| Peru model | 128 | 50 | 0.1 | OneCycleLR | 0.2 | 0.05 | BCE loss | 1.0 | Adam |
| Multi-regional limited model | 128 | 50 | 0.01 | OneCycleLR | 0.2 | 0.05 | BCE loss | 1.0 | Adam |
| Multi-regional complete model | 128 | 30 | 0.1 | OneCycleLR | 0.2 | 0.05 | BCE loss | 1.0 | Adam |

**Supplemental results**

**Table 2:** Training and validation dataset composition of the Ethiopia, Niger and Peru single-region models.

| **Split** | **Ethiopia Model** | **Niger Model** | **Peru Model** |
| --- | --- | --- | --- |
| **Training (Subjects)** | 490 | 490 | 490 |
| **Training (Images)** | 1481 | 1461 | 1576 |
| **Validation (Subjects)** | 123 | 123 | 123 |
| **Validation (Images)** | 370 | 365 | 394 |

**Table 3:** Training and validation dataset composition of the Limited and Complete Multi-regional models. Data are shown as numbers (proportion in %).

| **Split** | **Dataset Source** | **Limited Multi-regional Model** | **Complete Multi-regional Model** |
| --- | --- | --- | --- |
| **Training** | ETHIOPIA (subjects) | 163 (33.3%) | 8963 (74.0%) |
|  | NIGER (subjects) | 163 (33.3%) | 2661 (22.0%) |
|  | PERU (subjects) | 163 (33.3%) | 490 (4.0%) |
|  | **Total (subjects)** | **489 (100.0%)** | **12114 (100.0%)** |
|  | ETHIOPIA (images) | 495 (32.8%) | 44816 (80.3%) |
|  | NIGER (images) | 486 (32.2%) | 7882 (14.1%) |
|  | PERU (images) | 527 (34.9%) | 3130 (5.6%) |
|  | **Total (images)** | **1508 (100.0%)** | **55828 (100.0%)** |
| **Validation** | ETHIOPIA (subjects) | 41 (33.3%) | 2241 (74.0%) |
|  | NIGER (subjects) | 41 (33.3%) | 665 (22.0%) |
|  | PERU (subjects) | 41 (33.3%) | 123 (4.1%) |
|  | **Total (subjects)** | **123 (100.0%)** | **3029 (100.0%)** |
|  | ETHIOPIA (images) | 123 (32.7%) | 11204 (80.3%) |
|  | NIGER (images) | 123 (32.7%) | 1971 (14.1%) |
|  | PERU (images) | 130 (34.6%) | 783 (5.6%) |
|  | **Total (images)** | **376 (100.0%)** | **13958 (100.0%)** |

**Table 4:** Ethiopia, Niger, and Peru Test set composition. Note that all single-region and multi-regional models were tested on these test sets for fair performance comparison.

|  | **Ethiopia test set** | **Niger test set** | **Peru test set** |
| --- | --- | --- | --- |
| **Test Subjects** | 154 | 154 | 154 |
| **Test Images** | 463 | 452 | 505 |

**Table 5:** Best-fold Ethiopia-trained model performance (Metric ± 95% bootstrapped CI using 10,000 iterations) across different test sets. Binarization threshold = 0.20.

| **Metric** | **Ethiopia Test set** | **Niger test set** | **Peru test set** |
| --- | --- | --- | --- |
| True Prevalence (%) | 28.57 | 3.25 | 25.97 |
| Predicted Prevalence (%) | 34.42 [26.62, 42.21] | 54.55 [46.75, 62.34] | 96.10 [92.86, 98.70] |
| Specificity | 0.86 [0.80, 0.93] | 0.47 [0.39, 0.55] | 0.05 [0.02, 0.10] |
| Sensitivity | 0.86 [0.76, 0.96] | 1.00 [1.00, 1.00] | 1.00 [1.00, 1.00] |
| Precision | 0.72 [0.59, 0.83] | 0.06 [0.01, 0.12] | 0.27 [0.20, 0.34] |
| Accuracy | 0.86 [0.81, 0.92] | 0.49 [0.41, 0.56] | 0.30 [0.23, 0.37] |
| F1_score | 0.78 [0.68, 0.87] | 0.11 [0.03, 0.21] | 0.43 [0.33, 0.51] |
| AUROC | 0.94 [0.88, 0.98] | 0.80 [0.61, 0.93] | 0.76 [0.67, 0.85] |
| AUPRC | 0.90 [0.82, 0.95] | 0.11 [0.04, 0.28] | 0.55 [0.40, 0.72] |

**Table 6:** Best-fold Niger-trained model performance (Metric ± 95% bootstrapped CI using 10,000 iterations) across different test sets. Binarization threshold = 0.0002.

| **Metric** | **Ethiopia Test set** | **Niger test set** | **Peru test set** |
| --- | --- | --- | --- |
| True Prevalence (%) | 28.57 | 3.25 | 25.97 |
| Predicted Prevalence (%) | 75.97 [68.83, 82.47] | 24.03 [17.53, 31.17] | 29.22 [22.08, 36.36] |
| Specificity | 0.29 [0.21, 0.38] | 0.79 [0.72, 0.85] | 0.85 [0.78, 0.91] |
| Sensitivity | 0.89 [0.78, 0.97] | 1.00 [1.00, 1.00] | 0.70 [0.55, 0.83] |
| Precision | 0.33 [0.25, 0.42] | 0.14 [0.03, 0.26] | 0.62 [0.48, 0.76] |
| Accuracy | 0.46 [0.38, 0.54] | 0.79 [0.73, 0.86] | 0.81 [0.75, 0.87] |
| F1_score | 0.48 [0.39, 0.58] | 0.24 [0.06, 0.41] | 0.66 [0.53, 0.77] |
| AUROC | 0.73 [0.65, 0.82] | 0.97 [0.93, 1.00] | 0.84 [0.77, 0.91] |
| AUPRC | 0.50 [0.37, 0.67] | 0.63 [0.16, 1.00] | 0.69 [0.55, 0.82] |

**Table 7:** Best-fold Peru-trained model performance (Metric ± 95% bootstrapped CI using 10,000 iterations) across different test sets. Binarization threshold = 0.20.

| **Metric** | **Ethiopia Test set** | **Niger test set** | **Peru test set** |
| --- | --- | --- | --- |
| True Prevalence (%) | 28.57 | 3.25 | 25.97 |
| Predicted Prevalence (%) | 14.29 [9.09, 20.13] | 9.74 [5.19, 14.29] | 27.92 [20.78, 35.06] |
| Specificity | 0.96 [0.93, 0.99] | 0.92 [0.87, 0.96] | 0.95 [0.90, 0.98] |
| Sensitivity | 0.41 [0.26, 0.56] | 0.60 [0.00, 1.00] | 0.93 [0.83, 1.00] |
| Precision | 0.82 [0.64, 0.96] | 0.20 [0.00, 0.43] | 0.86 [0.75, 0.95] |
| Accuracy | 0.81 [0.74, 0.86] | 0.91 [0.86, 0.95] | 0.94 [0.90, 0.97] |
| F1_score | 0.55 [0.38, 0.68] | 0.30 [0.00, 0.55] | 0.89 [0.81, 0.95] |
| AUROC | 0.82 [0.74, 0.90] | 0.82 [0.56, 0.98] | 0.98 [0.96, 0.99] |
| AUPRC | 0.74 [0.62, 0.85] | 0.21 [0.04, 0.58] | 0.94 [0.88, 0.99] |

**Table 8:** Best-fold Limited multi-regional model performance (Metric ± 95% bootstrapped CI using 10,000 iterations) across different test sets. Binarization threshold = 0.25.

| **Metric** | **Ethiopia Test set** | **Niger test set** | **Peru test set** |
| --- | --- | --- | --- |
| True Prevalence (%) | 28.57 | 3.25 | 25.97 |
| Predicted Prevalence (%) | 31.82 [24.68, 39.61] | 1.30 [0.00, 3.25] | 31.17 [24.03, 38.31] |
| Specificity | 0.87 [0.81, 0.93] | 0.99 [0.98, 1.00] | 0.90 [0.84, 0.96] |
| Sensitivity | 0.80 [0.67, 0.91] | 0.20 [0.00, 0.67] | 0.93 [0.83, 1.00] |
| Precision | 0.71 [0.58, 0.84] | 0.50 [0.00, 1.00] | 0.77 [0.64, 0.89] |
| Accuracy | 0.85 [0.79, 0.90] | 0.97 [0.94, 0.99] | 0.91 [0.86, 0.95] |
| F1_score | 0.75 [0.64, 0.84] | 0.29 [0.00, 0.73] | 0.84 [0.75, 0.92] |
| AUROC | 0.90 [0.84, 0.95] | 0.81 [0.57, 0.98] | 0.97 [0.94, 0.99] |
| AUPRC | 0.85 [0.76, 0.93] | 0.32 [0.04, 0.78] | 0.91 [0.80, 0.98] |

**Table 9:** Best-fold Complete multi-regional model performance (Metric ± 95% bootstrapped CI using 10,000 iterations) across different test sets. Binarization threshold = 0.27.

| **Metric** | **Ethiopia Test set** | **Niger test set** | **Peru test set** |
| --- | --- | --- | --- |
| True Prevalence (%) | 28.57 | 3.25 | 25.97 |
| Predicted Prevalence (%) | 32.47 [25.32, 40.26] | 1.95 [0.00, 4.55] | 31.17 [24.03, 38.31] |
| Specificity | 0.91 [0.85, 0.96] | 0.99 [0.97, 1.00] | 0.92 [0.87, 0.97] |
| Sensitivity | 0.91 [0.81, 0.98] | 0.20 [0.00, 0.67] | 0.97 [0.92, 1.00] |
| Precision | 0.80 [0.68, 0.90] | 0.33 [0.00, 1.00] | 0.81 [0.70, 0.92] |
| Accuracy | 0.91 [0.86, 0.95] | 0.96 [0.93, 0.99] | 0.94 [0.90, 0.97] |
| F1_score | 0.85 [0.76, 0.92] | 0.25 [0.00, 0.67] | 0.89 [0.81, 0.95] |
| AUROC | 0.96 [0.92, 0.99] | 0.79 [0.48, 0.99] | 0.99 [0.97, 1.00] |
| AUPRC | 0.93 [0.87, 0.98] | 0.37 [0.03, 0.83] | 0.97 [0.92, 1.00] |

**References:**

1. Howard A, Sandler M, Chu G, et al. Searching for MobileNetV3. In: 2019:1314-1324. Accessed April 8, 2025. https://openaccess.thecvf.com/content_ICCV_2019/html/Howard_Searching_for_MobileNetV3_ICCV_2019_paper.html

2. Russakovsky O, Deng J, Su H, et al. ImageNet Large Scale Visual Recognition Challenge. *arXiv*. Preprint posted online January 30, 2015. doi:10.48550/arXiv.1409.0575

3. Smith LN, Topin N. Super-Convergence: Very Fast Training of Neural Networks Using Large Learning Rates. arXiv.org. August 23, 2017. Accessed April 15, 2025. https://arxiv.org/abs/1708.07120v3

4. Kingma DP, Ba J. Adam: A Method for Stochastic Optimization. *arXiv*. Preprint posted online January 30, 2017. doi:10.48550/arXiv.1412.6980
